# Rosin-Based Epoxy Vitrimers with Dynamic Boronic Ester Bonds

**DOI:** 10.3390/polym13193386

**Published:** 2021-10-01

**Authors:** Yanning Zeng, Jiawei Li, Shuxin Liu, Bin Yang

**Affiliations:** Key Laboratory of New Processing Technology for Nonferrous Metal and Materials (Ministry of Education), College of Material Science and Engineering, Guilin University of Technology, Guilin 541004, China; zjljw1998@163.com (J.L.); liushuxin@126.com (S.L.); a18832111836@163.com (B.Y.)

**Keywords:** rosin, vitrimer, reprocessing, self-healing

## Abstract

Rosin is an abundantly available natural product. In this paper, for the first time, a rosin derivative is employed as the main monomer for preparation of epoxy vitrimers to improve the mechanical properties of vitrimers. Novel epoxy vitrimer networks with dynamic reversible covalent boronic ester bonds are constructed by a reaction between thiols in 2,2′–(1,4–phenylene)–bis (4–mercaptan–1,3,2–dioxaborolane) (BDB) as a curing agent and epoxy groups in the rosin derivative. The rosin-based epoxy vitrimer networks are fully characterized by Fourier transform infrared spectroscopy (FTIR), an equilibrium swelling experiment, and dynamic mechanical analysis (DMA). The obtained rosin-based epoxy vitrimers possess superior thermostability and good mechanical properties. Due to transesterification of boronic ester bonds, rosin epoxy vitrimer network topologies can be altered, giving welding, recycle, self-healing, and shape memory abilities to the fabricated polymer. Besides, the effects of treating time and temperature on welding capability is investigated, and it is found that the welding efficiency of the 20% C-FPAE sample is >93% after treatment for 12 h at 160 °C. Moreover, through a hot press, the pulverized samples of 20% C-FPAE can be reshaped several times and most mechanical properties are restored after reprocessing at 200 °C for 60 min. Finally, chemical degradation is researched for the rosin-based epoxy vitrimers.

## 1. Introduction

Thermosetting polymers that originate from petroleum chemicals have been involved in many industrial applications, such as protective coatings, wind turbines, high-performance materials for aircrafts, and other areas, because of their dimensional stability, mechanical properties, and creep/chemical resistance [1]. However, the inherent irreversible cross-linking naturally restricts their flow and they cannot be reshaped, reprocessed, or recycled, leading to an aggravating petroleum resource crisis. To address this issue, dynamic covalent bonds can be incorporated into the thermoset polymer network, giving it the unique features, such as malleability, self-healing, and reprocessing properties. Vitrimers with dynamic covalent bonds flow when heated but remain insoluble [2]. A network of vitrimers can change their topologies without decreasing their connectivity, attributed to associative exchange reactions, which maintain constant the number of chemical bonds and cross-links. Moreover, vitrimers can flow when a stimulus is applied, because associative exchange reactions permit the network topology to fluctuate; furthermore, the kinetics change can control the relaxation dynamics and viscosity of vitrimers [3,4]. At high temperatures, vitrimers can be processed due to fast exchange reactions. At low temperatures, the shape is fixed either by quenching the exchange reactions [5] or by the motion of polymer chains through the glass transition temperature (*T_g_*) or crystallization [3,6]. Thus, vitrimers with the desired chemical properties can be reshaped and recycled. The initial epoxy vitrimer network is reorganized via transesterification [2]. Currently, the library of dynamic exchange reactions has expanded to include chemistries such as boronate ester exchange [7,8,9], boroxine exchange [10], dioxaborolane metathesis [8], transamination [11], trans-N-alkylation [12], reversible addition of thiols [13], imine exchange [14], olefin metathesis [15], and transcarbonation [16] Among these chemistries, boronate ester bonds are renowned for their dynamic and robust features and have been widely employed in solution-based systems for molecular sensors [17] and self-healing hydrogels [18]. In contrast, improvements in vitrimer mechanical performance are achieved at the expense of dynamic properties due to restricted chain mobility, and it is a challenge to enhance both mechanical performance and dynamic properties.

Rosin is an abundantly available and low-cost natural product and primarily consists of rosin acids with characteristic hydrophenanthrene structures and 10% neutral materials. Because of hydrogenated phenanthrene ring, rosin acids have cycloaliphatic structures, giving the features of excellent biodegradability, solubility, and biological compatibility. As a consequence, rosin and its derivatives have served as alternatives to petroleum-based rigid monomers in polymer fabrication [19,20], such as polyurethane [20], polyester [21], and epoxy resins [22,23]. Meanwhile, petroleum-based curing agents such as 1,2–cyclohexanedicarboxylic anhydride (CHDA) and 1,2,4-benzenetricarboxylic anhydride (BTCA) also can be replaced by rosin derivatives [19,22,24,25]. It is found that hydrogen phenanthrene ring structures of rosin acids are beneficial for the thermal and mechanical properties of the obtained thermosets. Moreover, *T_g_* improvements of the obtained cured acrylated epoxidized soybean oil are significantly influenced by the curing agent of rosin-based derivatives [26], and cured epoxies employing rosin as a curing agent display similar moduli and a higher *T_g_* than commercial monocyclic analogues [19,22]. Besides, polymers with rosin-based components, such as epoxy resin, polyester, and polyamide, exhibit high thermal stability or high mechanical properties [26,27].

To the best of our knowledge, there is not a similar study using rosin-based derivatives as the main monomer for vitrimer preparation to improve their mechanical properties in the literature. In this work, first, a rigid vitrimer monomer derived from rosin was synthesized, and then 2,2′–(1,4–phenylene)–bis(4–mercaptan–1,3,2–dioxaborolane) (BDB) as a curing agent reacted with the rosin-derived monomer by one-pot thermally initiated thiol-epoxy click chemistry, producing vitrimers with a framework of a rigid hydrogenated phenanthrene ring. The objective of this research is to make use of natural rosin to fabricate biobased vitrimers with high mechanical properties, thermostability, and *T_g_*. Meanwhile, we studied the fabrication of high-performance vitrimers using rosin resources, for vitrimers naturally have multifunctionality, such as self-healing, shape memory, and reprocessing.

## 2. Experimental Section

### 2.1. Materials

Rosin (acid number = 166 mgKOH·g^−1^) was kindly supplied by Guilin Xingsong Forest Chemical Co., Ltd (Guilin, China). 1–Thioglycerol (99%), 1,4–phenylenediboronic acid (97%), fumaric acid (FA), epichlorohydrin (EC), ethanol (EtOH), acetone, potassium hydroxide (KOH), triethylamine (Et_3_N), and 4–dimethylaminopyridine (99%) were purchased from Aladdin, Shanghai, China. Tetrahydrofuran (THF), toluene, dichloromethane, 30% hydrogen peroxide aqueous solution (H_2_O_2_), hydrochloric acid (HCl), and magnesium sulfate were purchased from XiLong scientific Co., Ltd (Shantou, China).

### 2.2. Synthesis of Fumaropimaric Acid (FPA)

FPA was synthesized according to a previous work [28]. Briefly, a 500 mL four-neck round-bottomed flask equipped with a gas tube, a mechanical stirrer, a thermometer, and a N_2_ inlet was charged with 200.0 g of rosin (0.66 mol rosin was considered a pure substance) and 38.4 g of FA (0.33 mol), purged with a N_2_ stream. The system was heated to 190 °C and maintained at this temperature for 1 h, after which another 38.4 g of FA (0.33 mol) was added. The reaction was kept at 190 °C for 6 h, and the resulting crude product was received. Then the crude products were removed from the flask, purified using the potassium salting-out method, and recrystallized from acetic acid. The products were collected and dried to obtain white crystals (yield: 83%).

### 2.3. Synthesis of Epoxy Resins (FPAE)

FPAE was synthesized according to a previous work [29]. Briefly, a 250 mL four-neck round-bottomed flask, equipped with a reflux condenser, a mechanical stirrer, a thermometer, and a N_2_ inlet, was charged with FPA (30 g, 0.073 mol), EC (122 g, 1.32 mol), and 0.15 g of triethylamine (0.1 wt % on the basis of the total weight of FPA and EC). The acidity of the system was monitored by titration with KOH ethanol solution. The temperature of the system was maintained at 110 °C until the acid number was less than 0.5 mg KOH/g. A total of 10 g of solid KOH (0.18 mol) was added to the reaction after the temperature was cooled to 60 °C, and maintained at 65 °C for 1 h. Another 6 g (0.11 mol) of solid KOH was then introduced into the system. The reaction was complete after the system was maintained at 65 °C for 3 h. Purification of the crude product was carried out via filtering to remove inorganic substances such as KCl and KOH. The filtrate was washed with water several times until pH = 7 was reached, after which the water phase was removed. Purification was continued by heating at 80 °C for 3 h to remove EC and H_2_O in a vacuum oven and obtain a yellow, transparent block product (yield: 78.5%; theoretical epoxy value: 0.51 mol/100 g; found: 0.46 mol/100 g).

### 2.4. Synthesis of C-FPAE Cross-Linking Network

**Figure 1:**
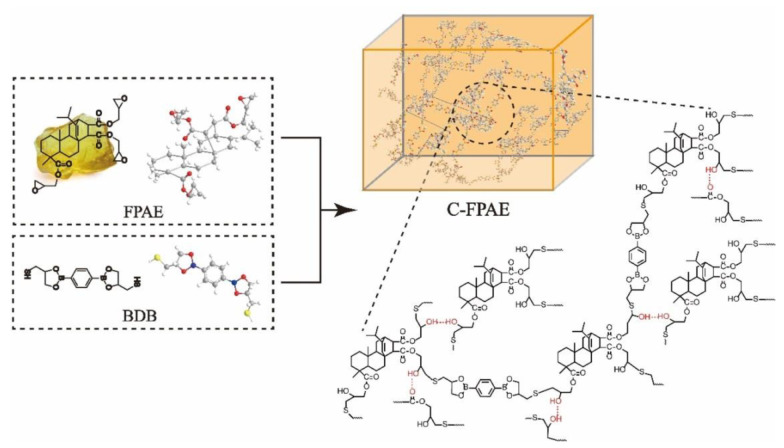
Synthesis of a C-FPAE cross-linking network.

A typical reaction of BDB synthesized as reported [30] and FPAE is showed in Figure 1 to obtain C-FPAE networks. FPAE (3.00 g, 5.20 nmol) in a three-necked flask was dissolved in THF. A catalysis amount of 4–dimethylaminopyridine and BDB (0.32 g, 1.04 nmol), which was synthesized as reported [31], were added under accelerating stirring until they dissolved. The mixture was quickly transferred to a release paper mold for 8 h at 120 °C to get cured FPAE (20% C-FPAE). BDB contents were 4.0, 8.0, 12.0, 16.0, 20.0, 50.0, and 100.0 mol% relative to FPAE, and the abbreviation of x% C-FPAE refers to cured FPAE with x mol% of BDB. However, the obtained 50%C-FPAE and 100%C-FPAE samples contained many bubbles, and even they were sequentially heated (50 °C, 2 h→80 °C, 2 h→100 °C, 2 h→120 °C 2 h) for curing, maybe due to the faster reaction at a high BDB content. Therefore, the properties of 50%C-FPAE and 100%C-FPAE could not be investigated.

### 2.5. Self-Healing, Welding, Shape-Memory, and Reprocessing

Self-healing experiments were recorded using a polarizing optical microscope (POM) equipped with a heating stage and a UCMOS05100KPA (P/N: TP605100A) microscope camera, Hangzhou, China. The film sample (20% C-FPAE) was cut using a razor to obtain cracks of comparable size. The cracked films were heated in an oven at 170 °C for 30 min, and thereafter, the cracks were observed using a POM.

Welding was performed with two rectangular samples of 20% C-FPAE (25 mm × 5 mm). They were held together with a superimposed length of 10 mm for holding times from 1~12 h at controlled temperatures (130, 140, 150, 160 °C). Good contact was ensured after the heating treatment, and welding efficiency was evaluated by conducting tensile tests at room temperature with a cross-head speed of 5 mm/min on the assembly and comparing the forces at break.

A strip sample (20% C-FPAE) was checked to study the shape memory capability. The strip sample was put into an oven at 130 °C, bent into different shapes using an external force, and finally cooled down to room temperature. Digital photos of the strip sample before and after reshaping were recorded.

The reprocessing sample (20% C-FPAE) was crushed and ground into a powder using a 0.25 mm aperture screen ring to obtain a powder with a particle size of 0.25 mm or less. A hydraulic plate vulcanizer (ZS-406B-30-300, Dongguan Zhuosheng Machinery Equipment Co., Ltd.Dongguan, China) was used as reprocessing equipment, and the obtained sample powder was added into the mold for reprocessing under 10 MPa pressure. Reprocessing was performed at 200 °C for 60 min, and the process was repeated for three cycles.

### 2.6. Degradation

Chemical degradation of the 20% C-FPAE samples was performed by immersing the powder samples (0.5 g) into a mixed solution of THF-H_2_O_2_-HCl with different pH (0.0, 0.2, 1.5, 3.7, 4.4, 6.5) at 30 °C for 48 h with stirring. The insoluble residuals were collected and vacuum-dried, and the degradation weight percentages were calculated. The soluble part was characterized by Fourier transform infrared spectroscopy (FTIR) and real-time Fourier transform infrared spectroscopy (real-time FTIR).

### 2.7. Characterization

FTIR spectra were collected using a Nicolet 205 FTIR spectrometer (Madison, USA) from 600 to 4000 cm^−1^ by the KBr tablet method. The thermal decomposition behavior of the FPAE and C-FPAE series was examined by means of thermogravimetry analysis (TGA) with a heating rate of 10 K/min in a nitrogen atmosphere from 35 to 800 °C on a TA Q500 (Milford, USA). Differential scanning calorimetry (DSC 204, NETZSCH, Germany) was performed at a heating rate of 5 °C/min from 25 to 300 °C in a nitrogen atmosphere. The glass transition temperature, *T_g_*, was obtained from the inflection point of the step of the curve by the thermomechanical method in the second cycle (heating to 200 °C). Dynamic mechanical analysis (DMA) and stress relaxation tests were carried out using a TA Q800 (Milford, USA) instrument. The C-FPAE samples (5 mm× 25 mm×1 mm) were tested from 25 to 180 °C (heating rate = 3 °C min^−1^) with a frequency of 1 Hz. Stress relaxation experiments were conducted by monitoring the stress decay at a constant strain of 1% after equilibrating at required temperatures for 20 min. The mechanical performance test used the UTM4503SLXY universal tensile testing machine of Shenzhen Sansi Aspect Technology Co., Ltd. (Shenzhen, China), with a 5 mm/min tensile rate. Young’s modulus, elongation at break, and tensile strength were obtained by the mechanical performance test. The length of the original sample for mechanical testing was 40 mm, and the length of the fractured sample was in the range of 40.5~42.4 mm. The density of the polymer was measured by a TWS series touchscreen electronic densitometer (TWS-300S, Mzkeyi Co., Ltd., Shenzhen, China) for calculation of cross-linking density. Real-time FTIR spectra were collected using a ReactIR 15 (METTLER TOLEDO, Zurich, Switzerland) spectrometer from 500 to 4000 cm^−1^ by a diamond probe. The shape memory performance of 20% C-FPAE was also examined by DMA. The sample with a rectangular shape (35 mm × 5 mm × 1 mm) was allowed to undergo two cycles at a temperature of 120 °C and a rate of 3 °C min^−1^, and a constant stress of 0.05 N was used to induce shape changing.

The sol fraction and swelling ratio were determined by an equilibrium swelling experiment based on the Flory–Rehner equation, as showin in Equations (1) and (2). It was conducted by immersing vulcanizate in toluene, THF, or acetone refluxing for 24 h, and then solvent was wiped with filter paper. The samples were weighed immediately and dried in a vacuum oven at 60 °C until the weight was constant. Three specimens were measured for each sample. Assuming that the initial mass was $m_{0}$, the mass after swelling was $m_{1}$ and the mass after drying was $m_{2}$, and the swelling ratio and sol fraction were obtained:

(1) $$\mathrm{Swelling}\;\mathrm{ratio}\;\mathrm{is}\;\mathrm{defined}\;\mathrm{as}=\frac{m_{1}-m_{2}}{m_{2}}$$

(2) $$\mathrm{Sol}\;\mathrm{fraction}\;\mathrm{is}\;\mathrm{determined}\;\mathrm{as}=\frac{m_{0}-m_{2}}{m_{0}}$$

The cross-linking density (*C_d_*) of the produced polymer was determined by the equilibrium swelling method with toluene as the solvent. The polymer was cut into five samples (20 mm × 10 mm × 0.6 mm), weighed, and immersed in a separate bottle containing 50 mL of toluene for 5 days. After equilibrium swelling was achieved, the sample was dried between the sheets and weighed again. The *C_d_* of the C-FPAE series was calculated by Flory–Rehner formula (Equation (3)) [32,33].

(3) $$C_{d}=-\frac{\ln\left[\left(1-V_{r}\right)+V_{r}+\chi V_{r}^{2}\right]}{V_{s}\left(V_{r}^{1/3}-V_{r}/2\right)}$$

(4) $$V_{r}=\frac{m_{0}/\rho_{0}}{m_{0}/\rho_{0}+\left(m_{1}-m_{0}\right)/\rho_{c}}$$

(5) $$\chi =0.487+0.228V_{r}$$

where *m*_0_ is the initial mass of the sample, *m*_1_ is the mass of the sample after swelling equilibrium, *ρ_c_* is the density of toluene, *ρ*_0_ is the density of the polymer, *V_r_* is the volume fraction of the polymer, the calculation formula is Equation (4), *χ* is the interaction parameter between solvent and polymer, the simplified calculation formula is Equation (5), and *V_s_* is the molar volume of the solvent.

## 3. Results and Discussion

### 3.1. Covalent Cross-Linking of O-CFER Using BDB

**Figure 2:**
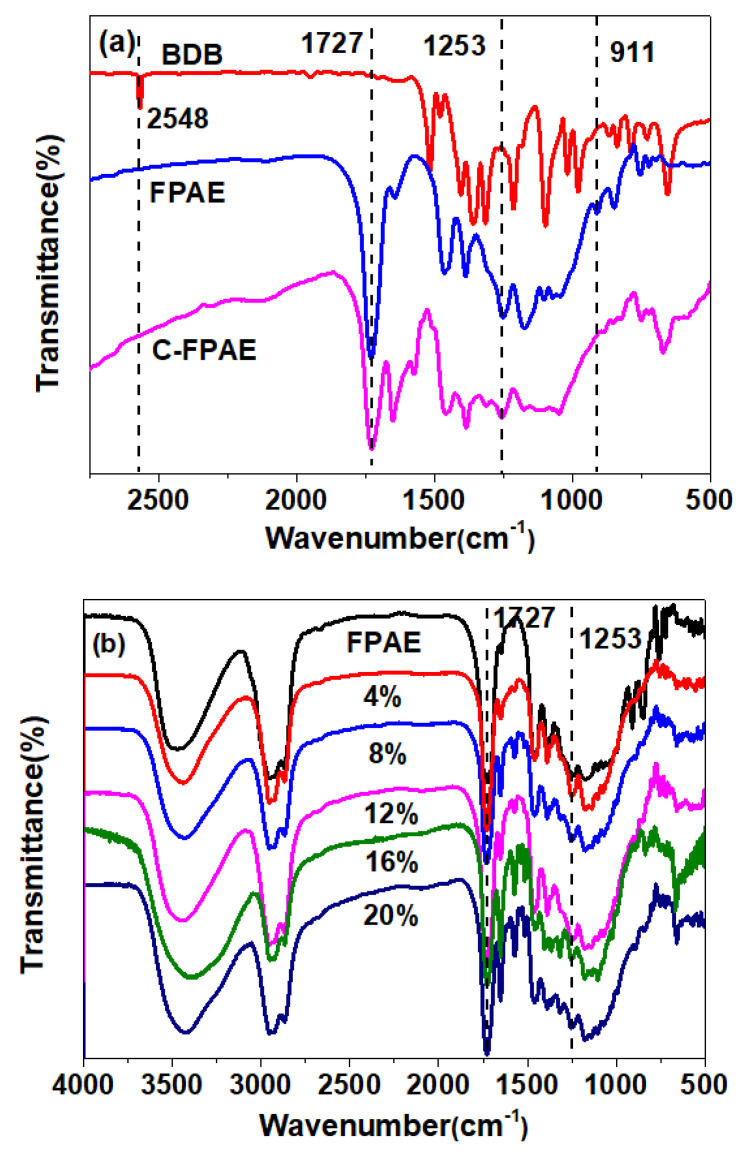

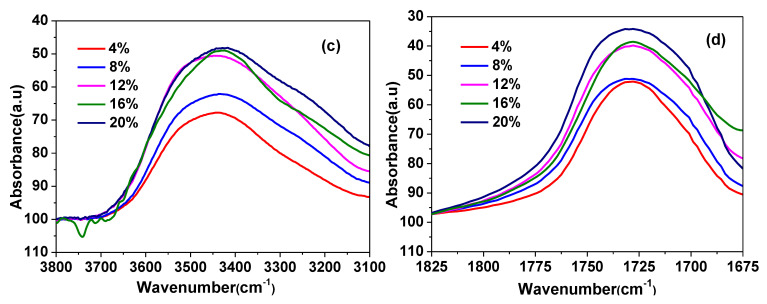
FTIR spectra of (**a**) BDB, FPAE, and 20% C-FPAE and (**b**) C-FPAE series with different BDB contents. (**c**) Hydroxyl and (**d**) carbonyl.

Boronic ester-containing C-FPAE networks were fabricated based on the chemical reaction between thiols of BDB and the epoxy groups of FPAE, with BDB as a curing agent. The fact could be explicitly confirmed by FTIR spectra in Figure 2a. In the spectrum of BDB, the absorption at 2548 cm^−1^ was ascribed to the stretching vibrations of –SH [34,35,36]. In the case of FPAE, the absorption peaks at 1253 and 911 cm^−1^ were attributed to the bending vibrations of the epoxy group [31]. The peak at 1727 cm^−1^ was attributed to the stretching vibrations of –C=O. Additionally, taking the absorption at 1727 cm^−1^ as a reference, the ratio of the peak intensity between 1727 and 1253 cm^−1^ (I(1727/1253) = 5.82 in Table 1) increased in cured 20% C-FPAE relative to FPAE (I(1727/1253) = 23.81) due to the reacted epoxy group, and with the BDB contents increasing (as Figure 2b), the value (I(1727/1253) increased from 6.50 to 23.81, indicating the more reacted epoxy group in 20% C-FPAE. Compared with the spectra of BDB and 20% C-FPAE samples, the absorption related to −SH in 20% C-FPAE completely disappeared after curing. Besides, the peaks at 1253 and 911 cm^−1^ present in the 20% C-FPAE spectrum indicated the existence of an unreacted epoxy group in the networks, while these two peaks obviously decreased in the 20% C-FPAE spectrum in comparison with those of FPAE. The above observations indicate the occurrence of a chemical reaction between thiols and epoxy groups. Moreover, the covalently cross-linked molecular architecture of the prepared C-FPAE sample can be further confirmed by the fact that it is insoluble in any organic solvent.

Hydrogen bonds are weaker than conventional covalent bonds but stronger than van der Waals forces, and it is believed that hydrogen bonds are present in the C-FPAE series. FTIR measurements were performed to verify the hydrogen bonds in the C-FPAE series with different BDB contents. As shown in Figure 2c,d), the peaks around 1717 and 3520 cm^−1^ were assigned to carbonyl and hydroxyl, and the two characteristic peaks were red-shifted, widened, or strengthened due to the formation of hydrogen bonds [36,37]. The absorption peaks at 3280 and 3401 cm^−1^ in Figure 2c were attributed to the intramolecular hydrogen bond O–H⋯O=C and the intermolecular hydrogen bond OH⋯O or OH⋯O=C, respectively [37,38,39,40], and the absorption peak shifted to lower wavenumbers when the BDB content increased from 4% to 20%, indicating more hydrogen bond formation at a higher BDB content.

**Figure 3:**
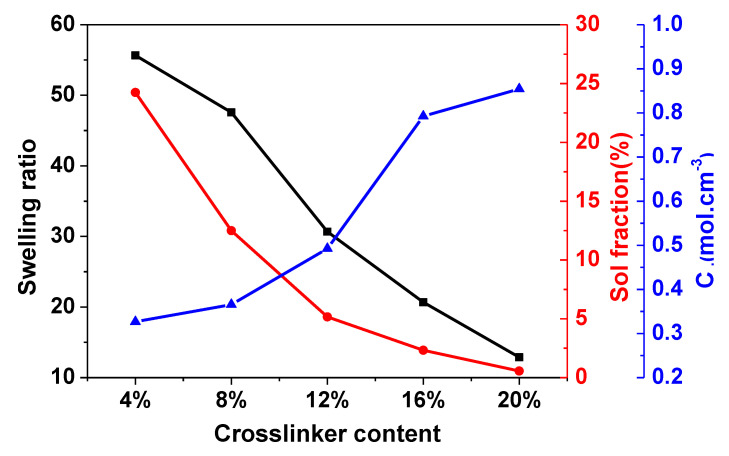
The cross-linking density, swelling ratio, and sol fraction of the C-FPAE series with different BDB contents.

Equilibrium swelling experiments showed that the cured C-FPAE series cannot be completely dissolved in toluene, THF, or acetone, and there was only a certain degree of swelling, which originated from the nature of a covalently cross-linked networks. The cross-linking density of the C-FPAE series consistently increased (0.33~0.85 mol·cm^−3^) with increase in the BDB content from 4% to 20% (Figure 3), revealing that a denser network is formed at a higher BDB content. Consequently, the sol fraction and swelling ratio monotonously decreased with the BDB load increasing [31,34].

### 3.2. Mechanical Properties, Thermal Performance, and Dynamic Property Analysis

**Figure 4:**
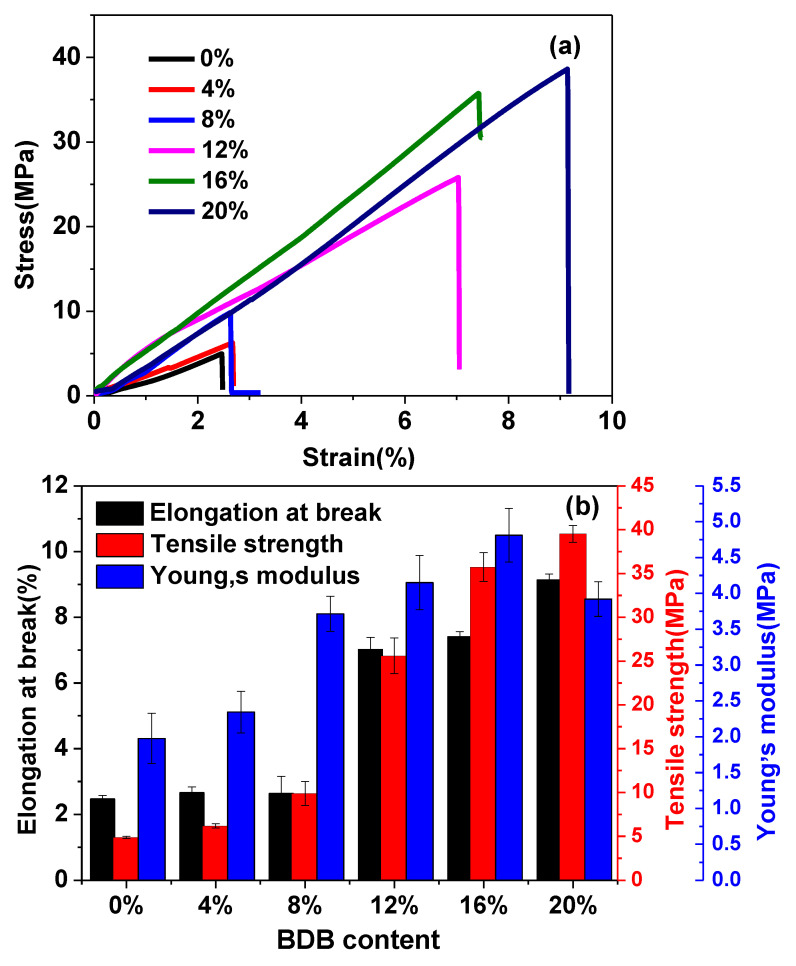
Mechanical properties of FPAE and C-FPAE series with different BDB contents: (**a**) stress–strain curve, (**b**) mechanical properties of the x% C-FPAE series with different cross-linker contents.

The representative stress−strain curves of the C-FPAE series with different BDB contents as well as pristine FPAE are shown in Figure 4a, and the mechanical properties are exhibited in Figure 4b. Pristine FPAE without cross-linking was stiff and relatively brittle, since the FPAE monomer includes epoxy and benzene groups. At a 2 mm/min strain rate, Young’s modulus was measured to be 1.7 MPa, with a tensile strength of 4.9 MPa and a strain at breaking of 2.5%. After curing by the BDB curing agent, the C-FPAE series was more ductile and tougher. Additionally, the mechanical properties could be facilely changed by varying the BDB content. Typically, increased BDB contents led to an enhancement in elongation at break and tensile strength as well as Young’s modulus. For instance, in comparison with those of FPAE, the Young’s modulus, elongation at break, and tensile strength of 20% C-FPAE increased from 1.7 to 3.9 MPa, from 2.5% to 9.1%, and from 4.9 to 39.5 MPa, respectively (Table 2). Increased BDB content brings out a more constrained network and restricted chain mobility due to increased cross-linking density. The mechanical properties of 20% C-FPAE were higher than those of epoxy vitrimers in the reported works (tensile strength was 10 vs. 4 MPa, elongation at break was 0.6% vs. 3.2%) [30,41]. It is noted that the Young’s modulus of 20% C-FPAE decreased with increasing BDB content. The origin of improved mechanical properties of the C-FPAE series should be the robust features of boronic ester bonds [31].

**Figure 5:**
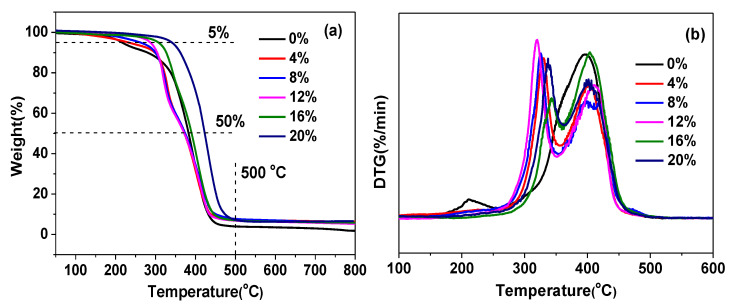
TGA (**a**) and DTG (**b**) curves of FPAE and C-FPAE series with different BDB contents.

The thermal stability of the C-FPAE series was researched by TGA at a heating rate of 10 °C/min under a nitrogen atmosphere, and the results are shown in Figure 5. Thermal stability factors, including initial decomposition temperature (the temperature of 5% weight loss, *T_5d_*) and the temperature of 50% weight loss (*T_50d_*) were determined by TGA. The values of *T_5d_*, *T_50d_*, and residual weight at 500/800 °C for the C-FPAE series are summarized in Table 3. It was observed that *T_5d_* and *T_50d_* of the C-FPAE series increased with increasing BDB content. The *T_5d_* of the C-FPAE series (BDB contents 4~20%) increased from 234.3 to 340.7 °C; however, the *T_5d_* of the FPAE monomer was only 214.5 °C. The residual weight at 500/800 °C was obviously enhanced by the introduction of the BDB curing agent due to the increased amount of sulfur and boron, and with the increasing BDB content, the thermal stability of C-FPAE prominently improved, and 20% C-FPAE showed the highest thermal stability (7.96% at 500 °C and 6.44% at 800 °C). The superior thermal stability of C-FPAE is attributed to the enhanced covalent cross-linker at higher contents of BDB, the improvement of the rosin framework, and the hydrogen bond in the networks [42,43].

**Figure 6:**
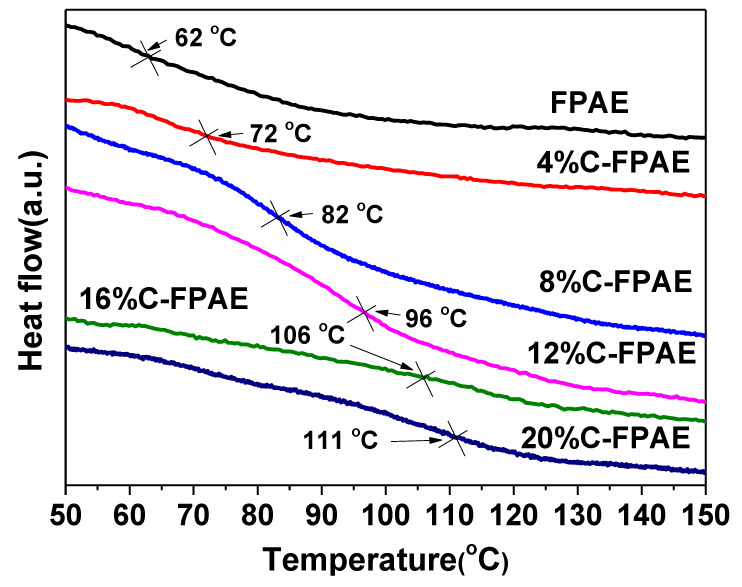
DSC curves of FPAE and C-FPAE series.

To adjust the welding and reprocessing performance of C-FPAE, *T_g_* is an important parameter. DSC curves are shown in Figure 6. In terms of the DSC curves in Figure 6, *T_g_* of the FPAE monomer was only 62 °C. As the BDB content increased from 4% to 20%, the *T_g_* of the C-FPAE series continuously increased from 72 to 111 °C, because with the BDB content increasing, more epoxy groups reacted and a higher cross-linking degree was achieved, giving a higher *T_g_* [44]. In addition, compared with other types of biobased thermosetting epoxy resins (*T_g_* lower than 80 °C), the C-FPAE series with rosin as a matrix displayed a higher *T_g_* [45,46]. With the aim of developing new epoxy resins that simultaneously show excellent welding and reprocessing capability, 20% C-FPAE having the maximum *T_5d_*, *T_50d_*, and *T_g_* was selected for evaluating the mentioned capability [3,47,48]. Besides, in terms of the *T_g_* (125 °C) of 20% C-FPAE, the temperatures of welding and reprocessing were set higher than 125 °C, expecting rapid shaping recovery and facile reprocessing.

**Figure 7:**
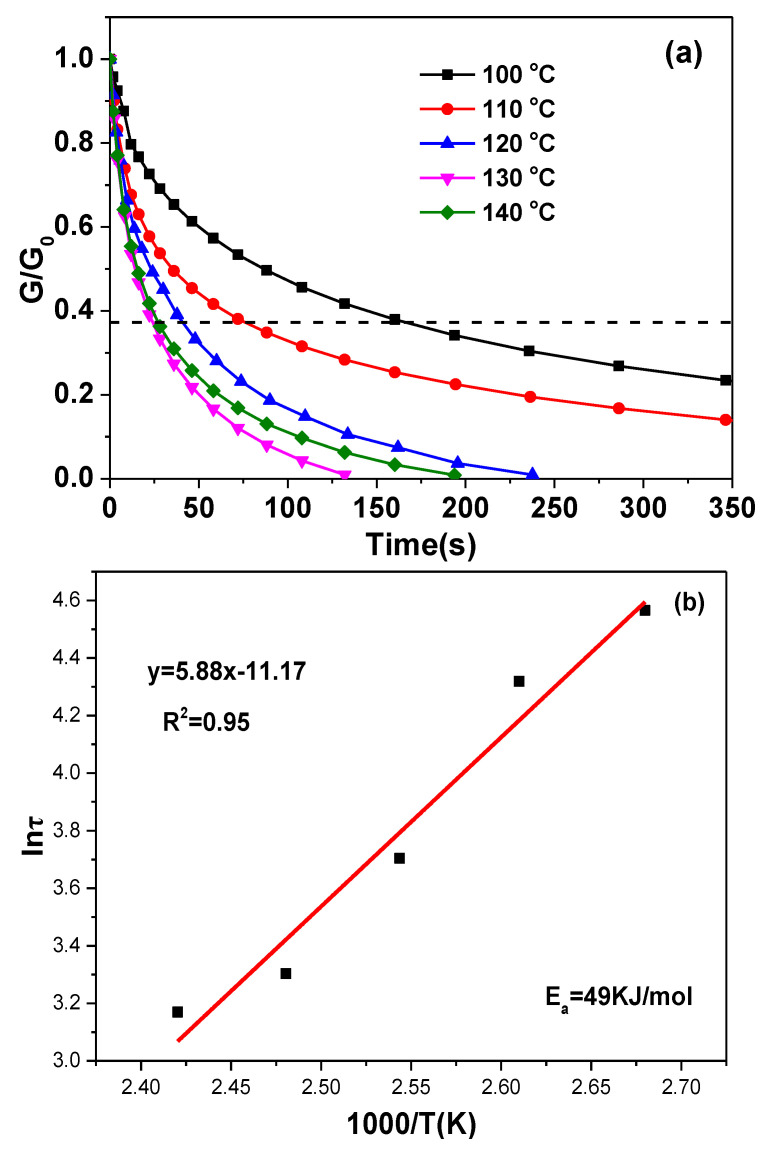
(**a**) Stress relaxation study of 20% C-FPAE. (**b**) Arrhenius plot with linear fit for 20% C-FPAE.

Stress relaxation experiments were conducted with DMA to evaluate the dynamic properties of the C-FPAE series. A deformation of 1% was used, and the decrease in stress was measured at different times but constant temperature (80, 100, 110, 120, 130, or 140 °C). Based on the Maxwell model for viscoelastic fluids, relaxation times were taken when the normalized stress decreased to 1/e of the initial stress. As shown in Figure 7a, the relaxation times decreased from 160 s at 100 °C to 24 s at 140 °C, and complete stress relaxation was accomplished above 120 °C. The temperature dependence of the relaxation time followed Arrhenius’ law (Figure 7b), which is given by Equation (6), and an activation energy *E_a_* of 49 kJ·mol^−1^ was obtained from the slope [3]. This result that the viscoelastic behavior of 20% C-FPAE follows Arrhenius’ law is the first to reveal rosin-based vitrimer materials.

(6) $$\;\ln\tau =\ln\tau_{0}+\frac{E_{a}}{RT}$$

The topology freezing transition temperature (*T_v_*) is a key parameter for vitrimers [6,49], and it corresponds to the transition from a solid to a viscous fluid. *T_v_* is conventionally defined as the temperature where the viscosity reaches 10^12^ Pa·s, and it was determined to be −31 °C in our system by extrapolating from the Arrhenius fitted line to a relaxation time of 4.8 × 10^5^ s. The borate exchange reaction has lower *E_a_* and *T_v_*, and the low *T_v_* should give a fast exchange reaction, consistent with its fast relaxation time at high temperature [10,50,51]. However, 20% C-FPAE displayed only slight stress relaxation at room temperature, since its network was frozen by the lack of segmental motions associated with a higher *T_g_*.

The effects of the BDB content on the α-relaxation and storage modulus of the C-FPAE series were studied. The plots of the storage modulus and tan δ for the C-FPAE series with different BDB contents are shown in Figures S1 and S2 (in Supplementary Materials). The typical tan δ plot of C-FPAE showed two loss peaks at round 61 and 125 °C (Figure S2). The two loss peaks are typical for polymers with different components [52,53,54]. The weak loss peak at 61 °C came from the incompletely cross-linked FPAE ingredients in the C-FPAE series. As the C-FPAE series warmed up, the main loss peak was found at 125 °C, as the second component of the macromolecular C-FPAE chain lost its mechanical integrity. The peaks around 125 °C of the tan δ curve became dominant with the increase in BDB contents, due to the sufficiently reacted epoxy group in C-FPAE. The *T_g_* (round 125 °C) was higher than that obtained by DSC due to different characterization methods. Additionally, the C-FPAE with a higher BDB content displayed a higher rubber platform (*E’*), and 20% C-FPAE showed the highest *E’* (61.9 MPa; Table 2), maybe because network integrity became much better at a BDB content of 20%, consisting of a tan δ plot of 20% C-FPAE with one dominate peak at 125 °C. This phenomenon should be related to the cross-linking density of the C-FPAE series, and the C-FPAE with a higher BDB content provides a more well-developed network structure, which restricts the chain mobility and thus enhances the elastic response of the network. Therefore, the enhanced storage modulus and the reduced peak height of the tan δ plot originate from the increasing BDB content of the C-FPAE series [52].

### 3.3. Self-Healing, Welding, and Shape Memory

**Figure 8:**
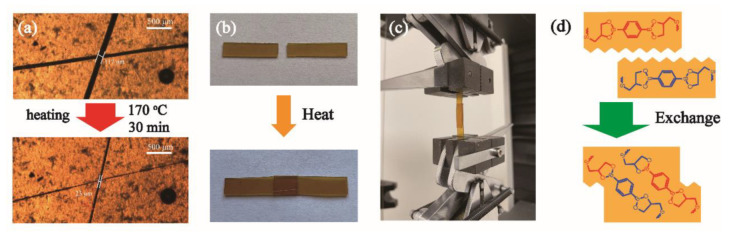
(**a**) Optical microscope images of self-healing performance for 20%C-FPEA. (**b**) Optical microscope images of welding performance. (**c**) Photograph of a sample during the lap-shear test. (**d**) Schematic representation of the transesterification reaction of boronic ester linkages.

Network rearrangement and bond reshuffling can take place, and covalent bonding can be re-established across the interfaces of fractured surfaces, because of the transesterification reaction of boronic ester linkages, as described in Figure 8d. As a result, 20% C-FPEA should acquire self-healing ability by boronic ester bond exchange-induced network rearrangement. The 20% C-FPEA sheet sample with a thickness of 0.7 mm was cut using a razor to a width of 117 µm to examine its self-healing capability. The cut sample had healing treatment at 170 °C for 30 min in the oven. As shown in Figure 8a, 20% C-FPEA exhibited good self-healing capability and only 30 min healing treatment led to more than 80 % recovery.

**Figure 9:**
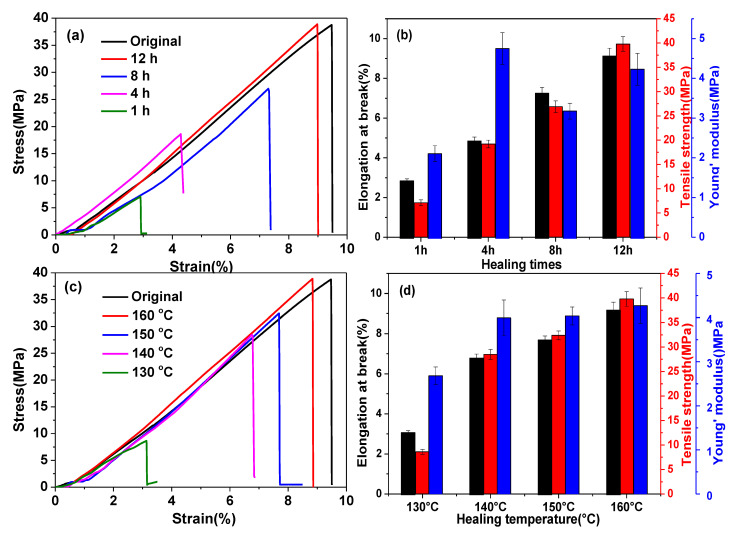
(**a**) Tensile curves of 20% C-FPEA welding at 160 °C for various times. (**b**) Mechanical properties of 20% C-FPEA welded at 160 °C for various times. (**c**) Tensile curves of 20% C-FPEA welding at 12 h for various temperatures. (**d**) Mechanical properties of 20% C-FPEA welded at 12 h for various temperatures.

To investigate the welding properties of 20% C-FPEA networks, the lap-shear test was used (Figure 8c), although the test is intrinsically difficult to pass for polymeric assemblies, as a fracture is easily initiated at the interface. Two rectangular samples of 20% C-FPEA (20.0 mm × 5.0 mm × 0.7 mm) were superimposed on an 8.0 mm length (Figure 8b) and held together under pressure for welding times ranging from 1 to 12 h at a controlled temperature (130, 140, 150, 160 °C). Remarkable contact was ensured by applying compression during the treatment. The mechanical properties of the welded samples were then evaluated by carrying out tensile tests at room temperature with a cross-head speed of 2 mm/min. We consider that the network could be frozen as the temperature decreased below *T_g_*, so we chose a temperature higher than *T_g_* as the reprocessing temperature. Stress−strain curves of assemblies of 20% C-FPEA with different heating times (1, 4, 8, 12 h) at 160 °C are shown in Figure 9a. With extended heating times, the mechanical properties of the assemblies gradually improved, and finally the highest mechanical properties were achieved by 12 h heating with elongation at break of 9.2%, tensile strength of 39.8 MPa, and Young’s modulus of 4.3 MPa. As expected, the mechanical properties of the welded samples increased by elevating the treating temperature (Figure 9d). When the treating temperature was elevated from 130 °C to 160 °C for 12 h, continuously improved mechanical properties were obtained. The mechanical properties sharply increased at 160 °C, and the stress−stain curve of 20% C-FPEA after welding at 160 °C almost overlapped with that of the pristine sample (Figure 9c). The present results demonstrate that 20% C-FPEA containing a dynamic cross-linking network possesses attractive thermal healing and welding capabilities.

**Figure 10:**
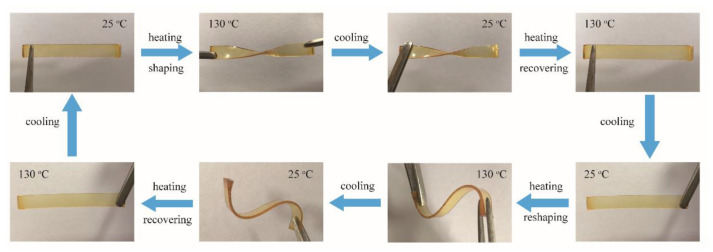
Optical microscope images of shape-memory performance of the 20% C-FPAE sample.

It is inherently difficult to reshape a typical cured polymer due to a permanent cross-linking network. Herein, the boronic ester undergoes an associative transesterification reaction in the network, which provides network rearrangement and contributes to a gradual Arrhenius-like viscosity dependence, giving the network the ability to be reshaped in a solid state. As a proof-of-concept, a strip-shaped sample of 20% C-FPAE was first heated to 130 °C in the oven and turned into an oblique shape that could be fixed when the temperature decreased to room temperature. When the temperature increased to 130 °C again, the shape of S reverted to its original flat state within 2 min and then recovered by following the same heating−cooling procedure. As a representative, Figure 10 shows digital photos of 20% C-FPAE before and after shaping. The shape change is reversible, and the process is repeatable, since the thermally induced dynamic exchange reaction is attributed to the reorganization of the cross-linked covalent network [55,56]. Moreover, shape memory cycles [57] were conducted by DMA (Figure S3 in Supplementary Materials). At the initial shape memory cycle, 20% C-FPAE displayed a shape fixation ratio (R_f_) of 86% and a shape recovery ratio (R_r_) of 59%.

### 3.4. Reprocessing

**Figure 11:**
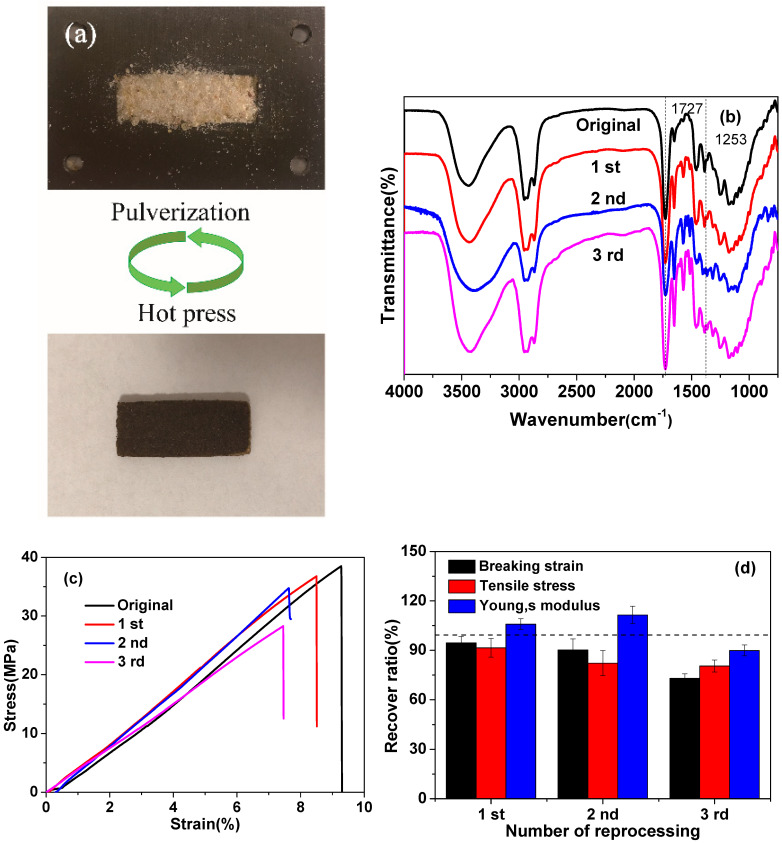
(**a**) Optical microscope images of thermal recycling performance of 20% C-FPAE by hot press. (**b**) FTIR spectra of 20% C-FPAE with different generations of reprocessing. (**c**) Tensile curves of samples with different generations of reprocessing. (**d**) Recovery ratios of samples with different generations of reprocessing.

In addition to the highly improved mechanical properties, shape memory capabilities and self-healing capabilities, the rosin vitrimer also demonstrates another smart property, reprocessability. The polymer networks of rosin vitrimers are only exchanged without consumptions during the recycling operation; therefore, several cycles might be achieved. To verify the reprocessability, the 20% C-FPAE sample was pulverized into a powder by using the same grade of sandpaper and then hot-pressed at 200 °C for 60 min, and homogeneous samples were obtained (Figure 11a). To quantify and qualify the reprocessability, the mechanical properties and FTIR measurement of the original and multiple recycled samples were examined. The intensity and wavelength of characteristic peaks at 1727 and 1253 cm^−1^ responding to –C=O and epoxy groups, respectively, in the FTIR spectra (Figure 11b) before and after several reprocessing cycles did not change, indicating similar chemical structures of the reprocessed and original samples and a high thermal stability of the C-FPAE series. Besides, recovery ratios of the mechanical properties of the recycled samples are shown in Figure 11d. It was observed that most mechanical properties were restored after reprocessing. For example, the recovery ratios of tensile strength, Young’s modulus, and elongation at break for the sample were 92%, 106%, and 95%, respectively. The stress–strain curves are shown in Figure 11c, and it is remarkable to note that even after reprocessing three times, the third-generation sample could lead to a tensile strength of 81% and a Young’s modulus of 90%. More strikingly, the samples could be repeatedly recycled because of the robust characteristic of the dynamic boronic ester bonds in the rosin vitrimer. Figure 11b shows the evolution of the ultimate stretch as a function of the number of time recycling was done. The properties of the recycled sample decay over the number of times recycling is done, while it stays in a reasonably good range, confirming that the boronic ester transesterification reaction plays a critical role in the reprocessing of rosin vitrimers [10].

### 3.5. Chemical Degradation

**Figure 12:**
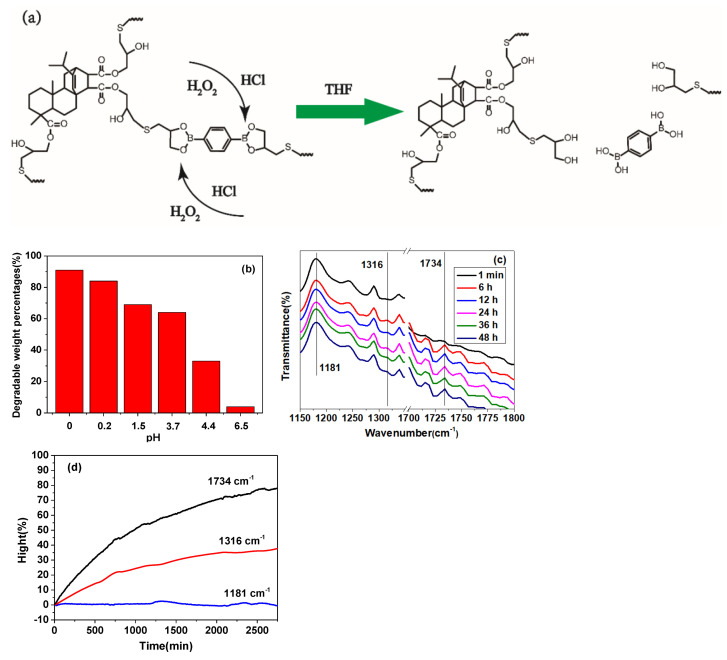
(**a**) Schematic representation of C-FPAE degradation. (**b**) The degradation weight percentage of 20% C-FPAE in mixed solutions with different pH. (**c**) Real-time FTIR spectra of 20% C-FPAE in mixed solution with pH = 0 for different times. (**d**) The height of the three characteristic peaks of FTIR (at 1734, 1316 and 1181 cm^−1^) during the degradation process.

Boronic ester bonds are highly sensitive to reactive oxygen species, and in acidic media with a pH much lower than their pKa, boronic ester bonds are more prone to hydrolysis and cleavage into boric acid and diol (Figure 12a) [58,59]. Due to unstable boronic bond under active oxygen and acidic conditions, degradability of the rosin vitrimer is expected. Here, 20% C-FPAE powder samples were immersed in a mixed solution of THF/H_2_O_2_/HCl with different pH at 30 °C for 48 h under continuous stirring, and the degradation weight percentages of the samples were calculated (Figure 12b). With the pH decreasing from 6.5 to 0.0, the degradable weight percentages increased from 10% to 90%. Therefore, we can conclude that a strong acid solution is desirable for 20% C-FPAE degradation. To investigate the degradation process, real-time FTIR was used, and Figure 12c shows the FTIR spectra of 20% C-FPAE at different degradation treating times (1 min, 6 h, 12 h, 24 h, 36 h, 48 h). The absorption peaks at 1181, 1316, and 1734 cm^−1^ originated from the stretching vibrations of –C–O–C– in the furan group, B-OH in the boronic acid bond, and C=O in carbonyl, respectively [60]. As the degradation time was prolonged, the absorption of B-OH and C=O gradually came out and became strong in the spectra. Meanwhile, the height of different peaks during the degradation process was recoded (Figure 12d). The increased absorptions at 1316 and 1734 cm^−1^ indicated the increase in the B-OH and C=O groups in the mixture solution, with stable absorption at 1181 cm^−1^ as reference. These results were attributed to the breakage of unstable boronic bonds, leading to a new generation of the B-OH group and soluble C=O group. This work first demonstrated the possibility of using boronic ester bonds to design degradable epoxy vitrimers.

## 4. Conclusions

A straightforward approach was established to fabricate a mechanically robust, weldable, malleable, and recyclable epoxy vitrimer, which are based on natural rosin having dynamic reversible boronic ester bonds. The networks of the rosin-based expoy vitrimer (C-FPAE) were obtained through chemical reactions between the thiols of BDB as a curing agent and the epoxy groups of the rosin derivative as a monomer, which were identified by FTIR spectroscopy and swelling experiments. High mechanical properties with a tensile strength of 39.5 MPa, elongation at break of 9.1%, and Young’s modulus of 4.2 MPa were obtained from 20% C-FPAE, and the mechanical behaviors could be regulated by changing the BDB contents from 4% to 20%. C-FPAE networks with reversibly covalent cross-linkers can rearrange their topology, allowing them be healed, welded, reshaped, and recycled, because of the dynamic reversible of boronic ester bonds. Furthermore, the C-FPAE sample recovered its original mechanical properties in the range of 70~115% after recycling. This work shows the potential of natural rosin for vitrimer network fabrication.

## Figures and Tables

**Table 1:** The ratio of peak intensity between 1727 and 1253 cm^−1^ of FPAE and C-FPAE with different BDB contents.

Sample	I (1727/1253)
FPAE	5.82
4% C-FPAE	6.50
8% C-FPAE	8.13
12% C-FPAE	11.37
16% C-FPAE	18.45
20% C-FPAE	23.81

**Table 2:** Mechanical and thermal properties of FPAE and C-FPAE with different BDB contents.

Sample	*T_g_* (°C)	E’(*T_g_* + 40 °C) (Mpa)	Elongation at Break (%)	Tensile Strength (Mpa)	Young’s Modulus (Mpa)
FPAE	62	/	2.5 ± 0.1	4.9 ± 0.1	1.7 ± 0.4
4%C-FPAE	72	3.2	2.7 ± 0.1	6.2 ± 0.2	2.4 ± 0.3
8%C-FPAE	82	4.1	2.7 ± 0.5	9.9 ± 1.0	3.7 ± 0.2
12%C-FPAE	96	5.5	7.0 ± 0.4	25.6 ± 2.0	4.2 ± 0.3
16%C-FPAE	106	7.8	7.4 ± 0.1	35.8 ± 1.6	4.8 ± 0.4
20% C-FPAE	111	61.9	9.1 ± 0.2	39.5 ± 1.1	3.9 ± 0.3

**Table 3:** Thermal stability factors of FPAE and C-FPAE obtained from TGA and DTG curves.

Sample	*T_5d_* (°C)	*T_50d_* (°C)	Residual Weight at 500 °C (%)	Residual Weight at 800 °C (%)
FPAE	214.5	381.9	3.98	1.87
4%C-FPAE	234.3	371.5	6.26	4.61
8%C-FPAE	259.5	372.3	6.72	5.07
12%C-FPAE	287.4	375.6	7.17	5.41
16%C-FPAE	307.3	390.4	7.63	5.98
20% C-FPAE	340.7	423.9	7.96	6.44

## Data Availability

Not applicable.

## Supplementary Materials

The following are available online at https://www.mdpi.com/article/10.3390/polym13193386/s1, Figure S1: Storage module curves; Figure S2: Tan δ curves of C-FPAE series from the DMA; Figure S3: Consecutive shape memory cycles curves of 20% C-FPAE; Table S1: The crosslinking density and other swell parameter of the C-FPAE series with different BDB contents.
