# Supplementary material for: Rosin-Based Epoxy Vitrimers with Dynamic Boronic Ester Bonds
Source: Polymers (Basel). 2021 Oct 1;13(19):3386. doi: 10.3390/polym13193386 (PMC8512039; doi:10.3390/polym13193386)
Supplement: Supplementary file 1 [file polymers-13-03386-s001.zip › polymers-1384431-supplementary.pdf]

polymers
MDPI

Supplementary materials

Rosin-Based Epoxy Vitrimers with Dynamic Boronic Ester Bonds

Yanning Zeng* Jiawei Li, Shuxin Liu and Bin Yang

Key Laboratory of New Processing Technology for Nonferrous Metal and Materials (Ministry of Education), and College of Material Science and Engineering, Guilin University of Technology, Guilin 541004, P. R. China
* Correspondence: Corresponding author: E-mail: ynzeng@glut.edu.cn

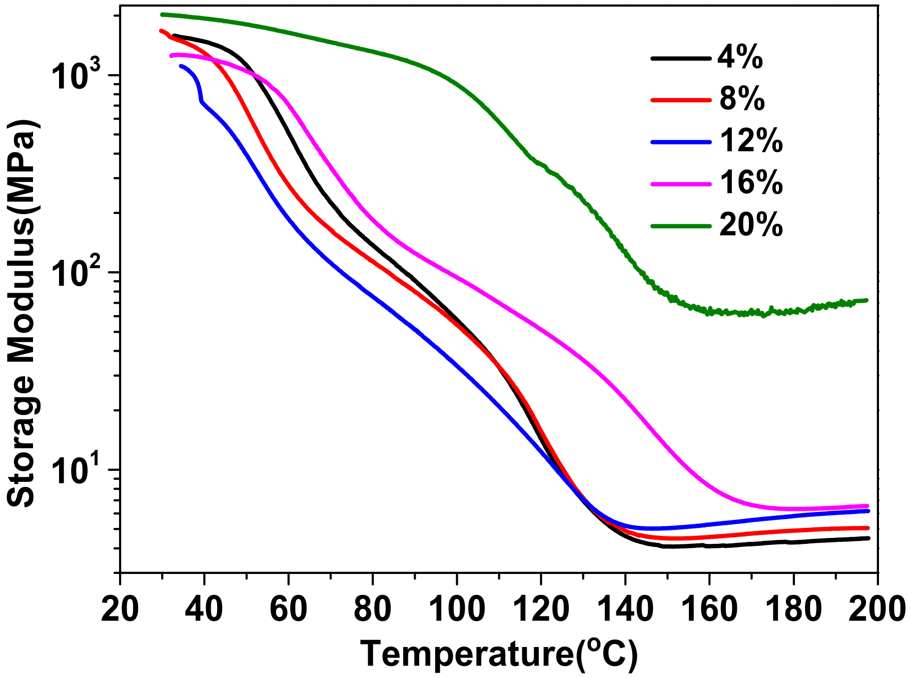

Figure S1 Storage module curves.

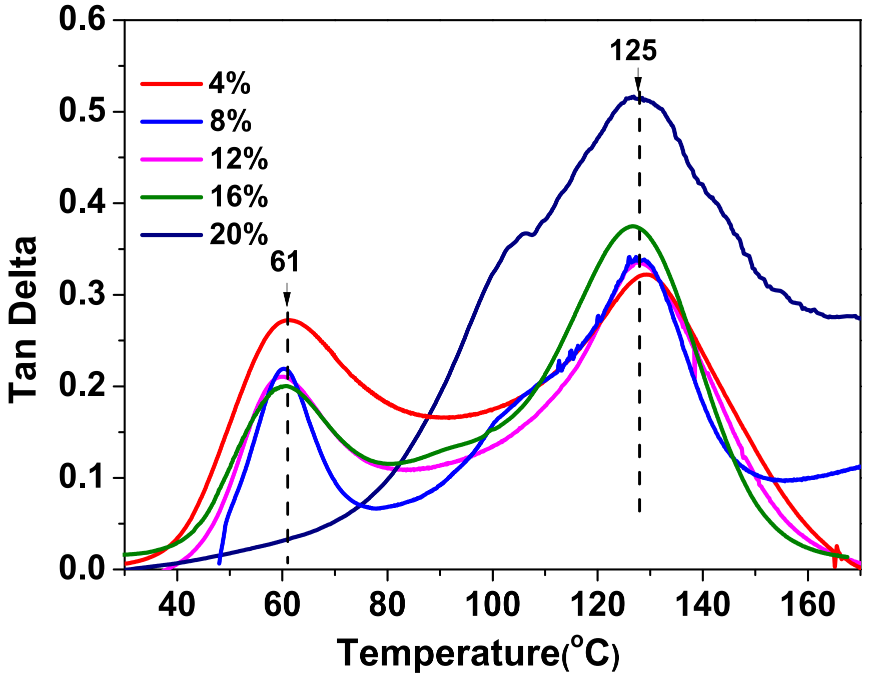

Figure S2 Tan δ curves of C-FPAE series from the DMA.

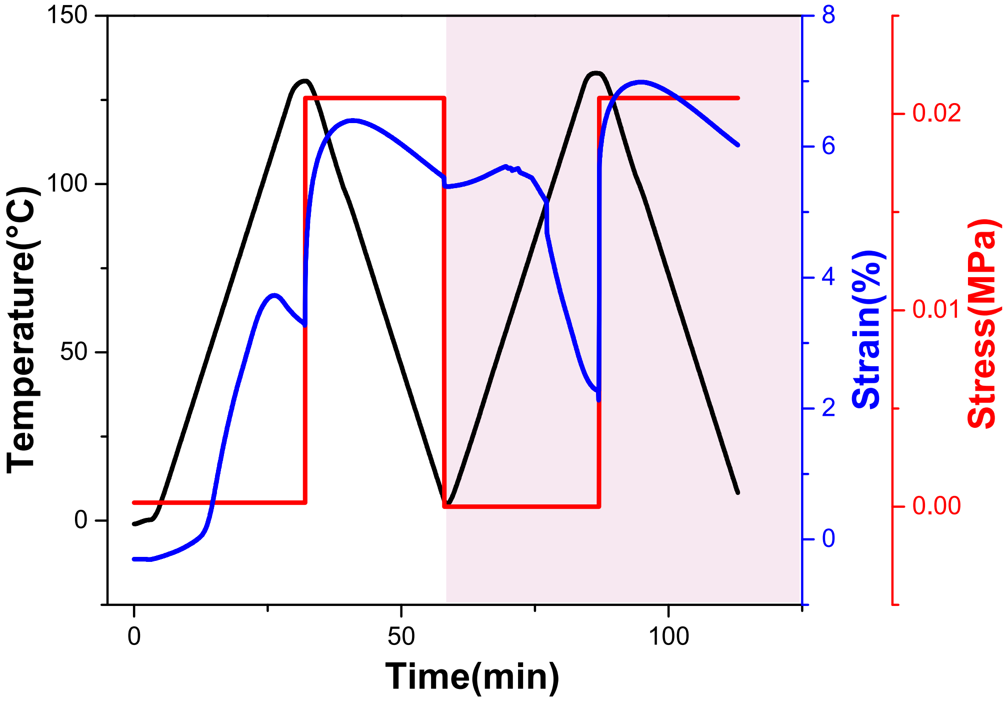

Figure S3 Consecutive shape memory cycles curves of 20% C-FPAE.

Shape fixation ratio (R_f) and shape recovery ratio (R_r) of 20% C-FPAE is defined as following equations. Where ε_1, ε_2 and ε_m are the strain after programming, residual strain after heating for shape recovery and maximum programming strain, respectively.

$$R_f = \frac{\varepsilon_1}{\varepsilon_m}$$

$$R_r = \frac{\varepsilon_1 - \varepsilon_2}{\varepsilon_1}$$

Table S1. The crosslinking density and other swell parameter of the C-FPAE series with different BDB contents

Sample	Swelling ratio	Sol fraction (%)	m_o (mg)	m_1 (mg)	ρ_0 (g/cm^3)	Cd (mol/cm^3)
4% C-FPAE	55.7	24.2	51.5	94.2	0.98	0.33
8% C-FPAE	47.8	12.5	83.2	114.8	1.02	0.37
12% C-FPAE	30.7	5.2	71.6	91.7	1.08	0.49
16% C-FPAE	20.7	2.3	463.1	573.3	1.15	0.79
20% C-FPAE	12.9	0.6	34.8	41.1	1.18	0.85

Article, Review, Communication, etc.)
